# Baboons, bacteria, and biological clocks address an age-old question

**DOI:** 10.7554/eLife.104715

**Published:** 2024-12-03

**Authors:** Amanda D Melin

**Affiliations:** 1 Department of Anthropology and Archaeology, University of Calgary Calgary Canada

**Keywords:** microbiome, biological clocks, aging, *Papio cynocephalus*, Amboseli Baboon Research Project, Other

## Abstract

Studying the fecal microbiota of wild baboons helps provide new insight into the factors that influence biological aging.

**Related research article** Dasari MR, Roche KE, Jansen D, Anderson J, Alberts SC, Tung J, Gilbert JA, Blekhman R, Mukherjee S, Archie EA. 2024. Social and environmental predictors of gut microbiome age in wild baboons. *eLife* **13**:RP102166. doi: 10.7554/eLife.102166.

“Please be prepared to show your ID” is a sign you will often find in liquor stores, bars and other establishments selling age-restricted products. Indeed, it can be exceedingly hard to accurately estimate someone’s chronological age, which is based on their date of birth, just by looking at them. However, whether an individual appears younger or older than they are is more than just a skin-deep matter.

Recent advances in molecular biology have identified indicators of ‘biological age’, an estimate of how old your cells and systems really are (Jylhävä et al., 2017). Understanding the genetic, social, and environmental factors that influence the rate at which the body of an individual gets older is a longstanding research goal with important implications for improving healthy aging (Oblak et al., 2021). By comparing biological and chronological ages, for example, it becomes possible to identify individuals who age more slowly and uncover the external elements favoring the persistence of youthfulness.

Common methods for assessing the biological age of individuals often rely on examining telltale signs of aging in the genetic material extracted from blood or tissue samples (Duan et al., 2022). However, recent studies have highlighted age-associated changes in the composition of the gut microbiome, the complex community of bacteria and other microorganisms that inhabit the intestine and influence many biological systems in the body. This has led scientists to successfully generate human ‘microbiome clocks’ based on key bacterial taxa (Chen et al., 2022). A great benefit of gut microbiome research is that it relies on a substance – poop – that is readily available and non-invasive to collect. This has opened many research avenues impossible to explore with more traditional approaches, particularly in animal studies.

The immense complexity of human lives greatly complicates the study of how social and environmental factors influence aging, and researchers often turn to animal models such as primates to overcome these limitations (Chiou et al., 2020). Baboons, for instance, share many similarities with humans when it comes to their genomes, physiologies, ecologies, behaviors, and social lives. By providing uninterrupted and detailed biological and behavioral insights into a wild population of yellow baboons (*Papio cynocephalus*) over several decades, the Amboseli Baboon Research Project has provided impactful contributions to the field (Alberts and Altmann, 2012). This includes helping to establish how social and physical attributes of an environment shape the composition and biological functions of the gut microbiome (Tung et al., 2015; Grieneisen et al., 2021). Now, in eLife, Mauna Dasari (University of Notre Dame and University of Pittsburgh), Elizabeth Archie (Notre Dame) and colleagues at various institutes in the United States, Germany and Canada report how baboon microbiome clocks can be established from fecal samples, and how these are influenced by various biological, social and ecological factors (Dasari et al., 2024; Figure 1).

**Figure 1.**
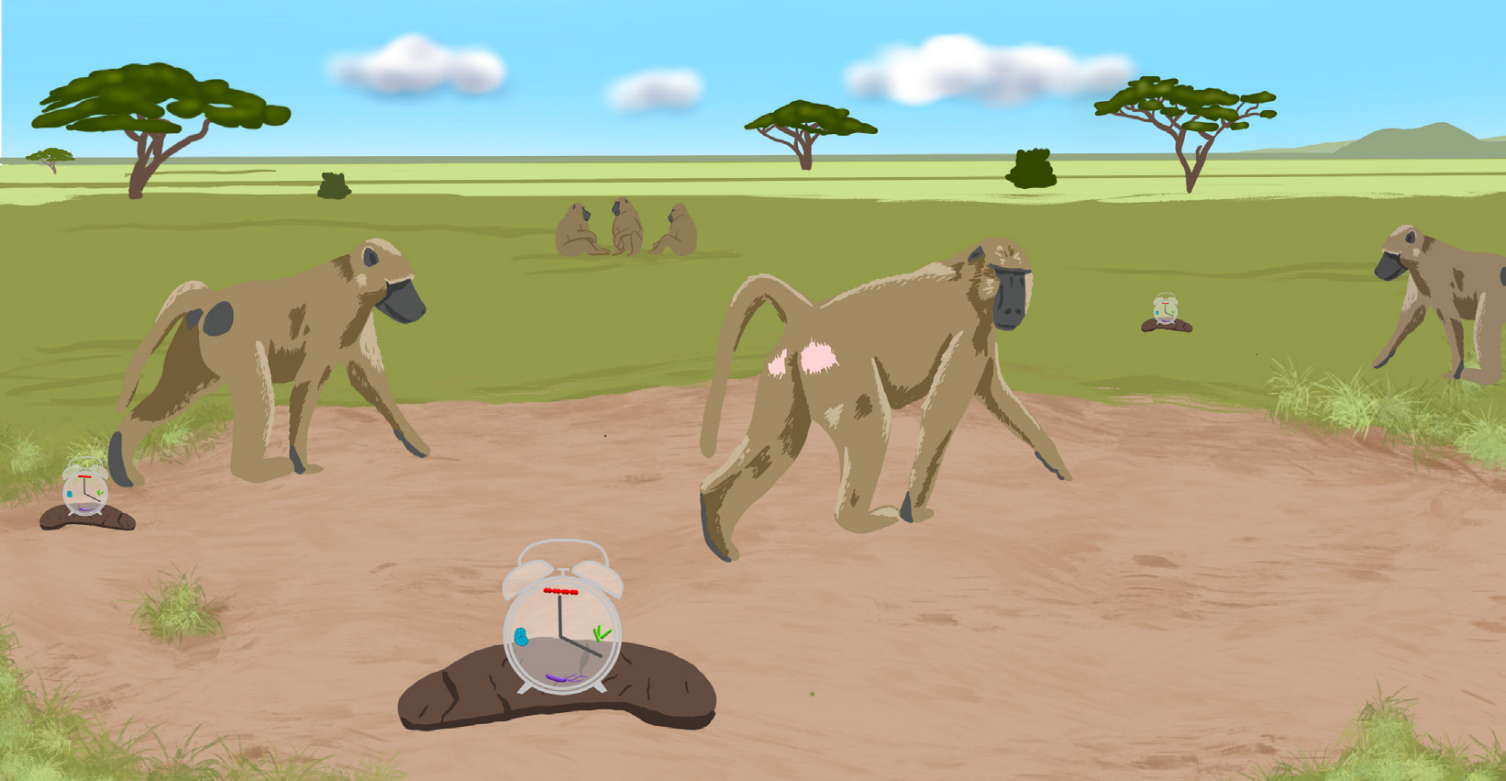
Baboon microbiome clocks can be established from fecal samples. Dasari et al. took advantage of data collected over several decades on a wild population of yellow baboons living in the Amboseli region to examine variation in biological age among individuals. They demonstrated that it was possible to use fecal samples to infer microbiome clocks based on age-related changes in the composition of various bacterial taxa (red, green, blue and purple icons within the clocks). In turn, these clocks made it possible to investigate the biological, social and environmental pressures that shape the pace at which an individual ages.

Image credit: Alyssa Bohart (CC BY 4.0).

The team used a supervised machine learning technique to predict the age of a baboon at the time of sampling based on the bacterial taxa present in its feces. The dataset included 13,563samples repeatedly collected from 479 baboons over 14years. While biological clocks established using information from the DNA of an individual remained the most precise, on average the fecal microbiome approach correctly estimated the chronological age of a baboon within about a two-year range, with the predictions for males being slightly better than for females. These results were more accurate than those obtained in the same population using some other aging biomarkers, such as body mass index or blood cell counts.

Clear sex differences in biological aging also emerged (a result consistent with previous studies on non-microbiome clocks), with sexually mature males being microbially older for their chronological age than females were. High-ranking males also had older microbiome clocks than their low-ranking counterparts, potentially due to the age-accelerating effects of the high energy requirements associated with having to physically fight to obtain and preserve their status. These results add to previous findings suggesting that dominant males are biologically old for their chronological age (Anderson et al., 2021). More unexpectedly, a similar pattern was also discovered in females, which tend to inherit their social rank rather than fight for it. The team predicted that low-ranking females, who experience more stressful conditions due to reduced access to food and other resources, would age more rapidly at a biological level – yet it was the higher-ranking females that had ‘older’ microbial features.

The gap between microbial and chronological ages varied greatly across baboons who, like humans, encounter differing experiences throughout their lives. For example, adverse events that take place early in life – such as losing a mother or living through extreme droughts as an infant – can have long-lasting consequences for development, aging and lifespan (Anderson et al., 2024). Counterintuitively, Dasari et al. found that early experiences of droughts or maternal isolation had no clear impact on females and were in fact linked to ‘younger’ microbiomes in males. This is especially surprising given that female samples collected during dry periods showed ‘older’ microbial signals than those obtained during the wet season, which presents fewer challenges for survival. Finally, none of the microbiome variables examined had predictive potential regarding mortality or an individual’s ability to attain major developmental milestones. Taken together, these findings indicate that periods of ecological stress can accelerate biological aging, but that current or recent conditions have a greater impact on biological age than those encountered early in life.

The work by Dasari et al. generates important insights into the processes influencing biological aging, and it is sure to inspire many future investigations, in particular into the potential impact of social networks. By showing that reliable biological clocks can be established using fecal microbiome analyses (and therefore easily accessible samples), this study may make it easier for researchers to investigate facets of aging within and across a variety of species.
